# Vital Spot of the Medulla Oblongata

**Published:** 1852-03

**Authors:** 


*Vital Spot of the Medulla Oblongata.* By M. FLOURENS.

At the meeting of October 27, of the Acad. Science of Paris, M. Flourens took occasion to speak of the actual locality of the vital spot, or primum mobile of the respiratory act. By several experiments he has now determined that that spot is situated exactly at the point of the calamus scriptorius, between the ventricle of Aurentius, and the junction of V-shaped gray matter of the pyramids. This spot is, according to M. Flourens, about the size of a *pin's head;* above or below the same a sharp instrument may be thrust in without causing the respiratory movements to cease; but when the exact spot is transfixed, life ceases immediately. M. Flourens had made this communication in order that the precise locality of the nodus vitæ should be well understood. — *London Medical Gazette.*

